# Application of a dried blood spot based proteomic and genetic assay for diagnosing hereditary angioedema

**DOI:** 10.1002/clt2.12317

**Published:** 2023-11-23

**Authors:** Marius‐Ionuţ Iuraşcu, Zsuzsanna Balla, Catarina Pereira, Noémi Andrási, Lilian Varga, Dorottya Csuka, Ágnes Szilágyi, Kornelia Tripolszki, Suliman Khan, Iuliana Susnea, Peter Bauer, Claudia Cozma, Henriette Farkas

**Affiliations:** ^1^ CENTOGENE GmbH Rostock Germany; ^2^ Department of Internal Medicine Hungarian Angioedema Center of Reference and Excellence Haematology Semmelweis University Budapest Hungary; ^3^ HNO‐Praxis Schaffhausen Schaffhausen Switzerland

**Keywords:** C1‐inhibitor, C4, DBS, HAE, SERPING1

## Abstract

**Background:**

Hereditary angioedema (HAE) with C1‐inhibitor deficiency (C1‐INH‐HAE) is a rare disease caused by low level (type I) or dysfunction (type II) of the C1‐inhibitor protein with subsequent reduction of certain complement protein levels.

**Methods:**

To develop and test the reliability of a two‐tier method based on C1‐INH and C4 quantitation followed by genetic analysis from dried blood spot (DBS) for establishing the diagnosis of C1‐INH‐HAE. C1‐INH and C4 proteins have been quantified in human plasma using a classical immuno‐assay and in DBS using a newly developed proteolytic liquid chromatography–mass spectrometry method. Genetic analysis was carried out as reported previously (PMID: 35386643) and by a targeted next‐generation sequencing panel, multiplex ligation‐dependent probe amplification and in some cases whole genome sequencing.

**Results:**

DBS quantification of C1‐INH and C4 showed the same pattern as plasma, offering the possibility of screening patients with AE symptoms either locally or remotely. Genetic analysis from DBS verified each of the previously identified *SERPING1* mutations of the tested C1‐INH‐HAE patients and revealed the presence of other rare variations in genes that may be involved in the pathogenesis of AE episodes.

**Conclusions:**

C1‐INH/C4 quantification in DBS can be used for screening of hereditary AE and DNA extracted from dried blood spots is suitable for identifying various types of mutations of the *SERPING1* gene.

## 1 BACKGROUND

Bradykinin‐mediated angioedema (AE) is a separate clinical entity characterized by recurrent AE attacks involving the subcutis and/or submucosa.^1^ It consists of two large groups of patients: AE with C1‐inhibitor (C1‐INH) deficiency and AE with normal C1‐INH function.^2^

The C1‐INH deficient group can be further divided into inherited and acquired diseases. Hereditary AE with C1‐inhibitor deficiency (C1‐INH‐HAE) is an autosomal dominantly inherited, rare disease caused by a mutation of *SERPING1* encoding C1‐INH. There are also two groups, C1‐INH‐HAE type I, which is approximately 85% of cases. This type is characterized by the production of reduced amounts of normal C1‐INH due to a heterozygous mutation resulting in impaired synthesis or secretion of the protein. Type II (15% of patients) is caused by a missense mutation, resulting in normal amount of C1‐INH protein but with defective function.^3^, ^4^

Acquired AE with C1‐inhibitory deficiency (C1‐INH‐AAE) is much less common than the hereditary form. The disease usually is secondary and caused by lymphoproliferative, other malignant, autoimmune or infectious diseases.^5^, ^6^, ^7^ Two pathomechanisms are hypothesized, one involves an increased consumption of the components of the classical complement pathway and C1‐INH, leading to increased bradykinin release through activation of the contact system. According to the other, autoantibodies against C1‐INH are formed, which neutralize C1‐INH.^8^, ^9^

The diagnosis of these diseases is based on laboratory testing of complement components. In the case of C1‐INH‐HAE, due to unrestricted activation, the levels of C2 and C4 will be decreased, while C1q and C3 will remain at normal levels. If the antigenic level of C1‐INH is abnormally low (usually less than 50% that would be expected for a well‐functioning allele) and this is accompanied by a decrease in the C1‐INH activity, a diagnosis of C1‐INH‐HAE type I is supposed. The Type II diagnosis is assumed if C1‐INH concentrations are normal or elevated, but C1‐INH activity is significantly decreased.^10^, ^11^ In the case of C1‐INH‐AAE, we find decreased levels of C4 and C1‐INH antigenic and functional activity, and unlike hereditary angioedema (HAE), C1q also shows a reduced value in the majority of these patients. Autoantibodies against C1‐INH (IgG, IgA, IgM) could be detected by ELISA. A diagnosis based on complement results can be supplemented by genetic testing of the *SERPING1* gene. To date, more than 800 pathogenic variants have been described in the gene, appr. 5% of which are derived from de novo mutations.^12^ Antigenic C1‐INH and C4 can be measured by radial immunodiffusion, nephelometric measurement of antigen‐antibody complexes, turbidimetry, or ELISA. Radial immunodiffusion, ELISA, or enzyme inhibition with C1s can also be used to measure C1‐INH functional activity.^13^, ^14^

Appropriate laboratory tests for the diagnosis of AE are usually performed on blood samples. Serum or plasma samples are required to complement tests, while Ethylenediaminetetraacetic acid (EDTA) anticoagulant blood is required for molecular genetic testing. Measurement of C1‐INH function should be performed in citrate anticoagulated plasma samples. Serum samples should be stored and shipped at 20–25°C and immediately centrifuged. It is recommended that complement tests be performed at accredited centers. Transporting a blood sample for testing is complicated and time consuming. If delivered within 24 h, native blood should be sent at room temperature. However, if transport is performed beyond 24 h, the blood samples should be centrifuged at the blood collection site. The centrifuged whey should be stored at least −20°C and transported to the laboratory on dry ice.^15^

Dried blood spot (DBS) has recently gained momentum as the preferred material type over liquid, blood‐derived samples due to their key advantages regarding collection, storage and transport.^16^, ^17^ A functional C1‐INH activity assay has been developed for DBS and it shows the increased stability of DBS samples (134 days at room temperature) over liquid blood (7 days at 4°C).^18^

Following our experience with DBS based assays we decided to develop an in‐house multiple reaction monitoring (MRM) mass spectrometric assay for the quantification of C1‐INH/C4 proteins that can be used for screening HAE patients in a high throughput environment. In the meantime, other DBS‐based assays have been reported but using a different mass spectrometric method.^19^

The aim of this study was to propose a two‐tier diagnostic scheme using dried blood spots based on proteolytic quantification of complement proteins followed by genetic confirmation using the same sample. For this purpose, the in‐house developed C1‐INH/C4 quantification assay from DBS was compared to the classical antigen tests in plasma in order to assess its applicability as a screening tool for HAE disease. For evaluating the reliability of genetic diagnosis of HAE from DBS samples a group of previously genotyped HAE patients with known mutations have been analyzed.

## 2 METHODS

### 2.1 Medical/clinical description of the patients

C1‐INH deficient patients (Supplementary Table S1) treated in the Hungarian Angioedema Center of Reference and Excellence (HACRE) appeared at least once a year in a follow‐up study to record the number and type of HAE attacks in the past year based on the annual patient diary, hospital reports, and outpatient data. In addition, complement levels (classical total complement, C3, C4, C1‐INH concentration level, and C1‐INH functional activity) were determined from blood samples taken from patients. Both AE symptoms and complement parameters were recorded simultaneously in the National AE Registry.

For this study, samples from patients were collected on an annual follow‐up visit. EDTA‐anticoagulated whole blood was transferred to DBS filter paper (CentoCard®, Centogene GmbH) according to the manufacturer's instructions.

The study protocol was approved by the institutional review board of Semmelweis University of Budapest, and informed consent was obtained from the participants in accordance with the Declaration of Helsinki.

The development of a new quantification method of C1‐INH and C4 from DBS was performed in the Centogene laboratory using samples from the Centogene DBS archive from genetically diagnosed patients with written consent for research use. The genes selected for this study were *SERPING1* for HAE I/II, *TTR* for Amyloidosis, *GLA* for Fabry disease, *GBA* for Gaucher disease and *GAA* for Pompe disease. A group of healthy donors with no AE symptoms was used as the control cohort.

### 2.2 Biomarker characterization

#### 2.2.1 Complement measurements from plasma

Diagnosis of C1‐INH‐HAE patients was established taking into account the level of complement C1‐INH, C4 and C1q, the functional activity of C1‐INH, as well as the presence of autoantibodies against C1‐INH. To quantify antigenic C1‐inhibitor levels, in‐house radial immunodiffusion was performed. The activity of C1‐inhibitor was measured using a C1‐inhibitor enzyme immunoassay kit (Quidel, San Diego, CA). The concentrations of C1q and autoantibodies against C1‐INH were determined by in‐house sandwich ELISA methods.^20^, ^21^ CH50 or total hemolytic activity of the classical pathway was determined using a hemolytic assay. C3 and C4 concentrations were measured by turbidimetry (Cobas Integra 400 analyzer, Roche, Switzerland). The clinical data and laboratory parameters of these patients were recorded in the National AE Registry. All experiments were performed in the Department of Internal Medicine and Hematology, Research Laboratory.

#### 2.2.2 Quantification of C1‐INH and C4 from dried blood spot

Complement proteins were quantified in dried blood spots using a newly developed method for the diagnosis of hereditary AE based on a quantitative mass spectrometric analysis as described in detail in the results section.^22^ Synthetic peptides were obtained as custom synthesized peptides from Peptide Specialty Laboratories (PSL) GmbH (Heidelberg, Germany). Leucine‐Enkephalin was obtained from Waters (Eschborn, Germany). Trypsin was purchased from Promega (Walldorf, Germany). All experiments were performed in the Centogene laboratories.

#### 2.2.3 Dried blood spots method development

Four proteins are associated with HAE Complement Cascade: plasma protease C1‐inhibitor, complement C1q, complement C3 and complement C4. Theoretical tryptic digestion was performed using Expasy (https://web.expasy.org/peptide mass/) and a list of all tryptic peptides was generated. A DBS sample from a healthy person was used to perform in solution tryptic digestion. Optimization was performed to obtain a complete digestion of the targeted proteins in dried blood spots. After purification, the sample was measured on a high resolution Vion IMMS‐QToF instrument (Waters, Eschborn, Germany) with an untargeted method. A library of peptides was generated from the theoretical tryptic digestion and used to scan for the presence of peptides in the digestion mixture. All the peptides detected in the sample were purchased as custom synthesized peptides from an external supplier (PSL). The synthetic peptides were used to create an MRM analysis method on AB‐Sciex 6500 triple‐quad mass spectrometer coupled with I‐class UPLC from Waters. Three pooled samples from a healthy individual, HAE type I and HAE type II pathological patients were used for peptide evaluation. Although all unique peptides are useable for the quantification of the proteins, we aimed at choosing the ones with the highest signal to noise ratio in our LC/MS system to better differentiate between the cohorts.

Two peptides were chosen for further assay establishment and validation: SERPING1 [^242^TLYSSSPR^249^] for the quantification of plasma protease C1‐inhibitor protein (C1‐INH), and C4Alpha [^680^NVNFQK^685^] for the quantification of complement C4 (C4). A cut off for each protein has been established to differentiate between healthy and pathological persons.

Sample preparation: three discs per sample (3.1 mm in diameter) were cut from the DBS filter paper (CentoCard®) in a 96 well plate using a PerkinElmer DBS puncher and transferred in 1.5 mL reaction tubes. 100 μL extraction buffer (90 mM ammonium carbonate buffer pH 7.5 with 0.012% (w/v) Na Taurocholate) were added to the DBS. The extraction was performed by 10 min sonication at 55°C, 30 min incubation at 37°C, shaking at 700 RPM, 10 min sonication at 55°C. The disulfide bridges were reduced using 12.5 μL 1M DTT solution and by incubation for 2.5–3 h at 37°C and 700 rpm, followed by alkylation with 37.5 μL 1M IAA solution and incubation for 1.5 h at 37°C and 700 rpm. Before proteolytic digestion, the pH was adjusted using 15 μL ammonium bicarbonate buffer (50 mM, pH 9.0) and 30 μL of trypsin solution were added to each sample and incubated for 16–18 h at 37°C and 700 rpm. The proteolytic reaction was stopped by changing the pH using the internal standard solution (2 μg/mL LeuEnk in acetonitrile:water:formic acid, 2:2:1, v:v:v). Before measurement, the samples were filtered using a PALL‐8031 filter plate on top of a 96 well plate and centrifugation for 5 min at 35,000 rpm.

LC/MS method: the samples were measured using a Waters Acquity UPLC (Waters GmbH, Eschborn, Germany) coupled with an ABSciex 5500 TripleQuad mass spectrometer (AB Sciex Germany GmbH, Darmstadt, Germany). The chromatographic run was performed on an ACE C8 column with pore size of 3 μm ((MZ‐Analysentechnik GmbH, Mainz, Germany) preheated at 60°C. The samples were separated using as solvents 50 mM FA in water (A) and 50 mM FA in methanol:acetonitrile 1:1 v:v (B), a flow rate of 0.5 mL/min and a linear gradient from 0% to 70% B solvent from min 2 to min 10. Upstream from the UPLC, a 3:1 flow splitter was added and a diverting valve (flow was diverted to the waste for the first 2 min of the run and starting with the minute 8.5 of the LC run). MRM‐MS analyses were performed in positive ion mode using the following parameters: CUR gas 25 psi, IS voltage 5.5 kV, CAD 8 psi, cone temperature 500°C, GS1 20 psi, GS2 20 psi, EP 10 V. Analysis and quantification were performed using the Analyst 1.6.2 software (AB Sciex Germany GmbH, Darmstadt, Germany) and MS Excel (Microsoft, Redmond, WA, USA) by making the average of the three analytical replicates.

The screening scheme for HAE patients done by monitoring C1‐INH and C4 in dried blood spots is the following: (i) if C1‐INH and C4 are normal, the person is healthy, (ii) if C1‐INH is normal and C4 is low, the patient may suffer from HAE Type II, (iii) if both C1‐INH and C4 are low, the patient may suffer from HAE Type I or Acquired AE, (iv) if C1‐INH is low and C4 borderline normal, exceptionally this might be a HAE case.

#### 2.2.4 Assay validation/characterization according to CAP/CLIA guidelines

The assay was validated in accordance with the CAP (College of American Pathologists) and CLIA (Clinical Laboratory Improvement Amendments) guidelines for best lab practices.

For sample quantification, a calibration line with concentrations between 0 and 500 nM was used with a linearity factor *R* ^2^ between 0.976 and 0.999. Sample injection volume proved to have a linearity factor *R* ^2^ between 0.994 and 0.987 for a range of 1–5 μL. The limit of quantification (LOQ) was determined to be 11 nM for C4 and 28.9 for C1‐INH, respectively, and the carry‐over below the LOQ.

The pathological range for C1‐INH was determined using 34 HAE Type I patient samples. For the C4 pathological range, 40 patient samples (HAE Type I and II) were used. The cut‐off was determined using the Mean + 3 * STD formula, resulting in 150 nM for C1‐INH and 210 nM for C4. When comparing the pathological cohorts with a group of 36 control samples, these cut‐off values gave both a sensitivity and a specificity of 100% for C1‐INH, and 95% for C4, respectively. The specificity of the assay was tested by comparing the same HAE Type I and II samples with a group of 17 non‐Angioedema patients suffering from other diseases (genetically confirmed): 5 Amyloidosis (TTR), 4 Fabry (GLA), 4 Gaucher (GBA) and 4 Pompe (GAA) patients. Both peptides showed values above the established cut‐offs, proving a specificity of 100%.

The inter‐ and intra‐assay precision and accuracy of the C4 and C1‐INH quantification showed CV values below 10%. For assay robustness the DTT reduction time was chosen resulting in CV below 3% for C4 and below 9% for C1‐INH. The stability of the assay was tested by storing the DBS for 0–4 weeks at various temperatures (−20, 4, 25, RT, 30 and 37°C) having the resulting CV below 9% for C4 and below 12% for C1‐INH.

### 2.3 Genetic analysis

#### 2.3.1 Genetic methods performed from DNA isolated from blood

In the frame of the routine molecular genetic diagnostics at the HACRE, DNA samples were isolated from EDTA‐anticoagulated blood samples and the entire coding region of the gene encoding C1‐INH (*SERPING1*; OMIM# 606860) was analyzed by direct bidirectional DNA sequencing following PCR amplification. The presence of copy‐number alterations (deletions or duplications of *SERPING1* exons) was detected using the multiplex ligation‐dependent probe amplification (MLPA) approach with the SALSA MLPA P243 probemix (MRC‐Holland). For detecting large deletions, long‐range polymerase chain reactions (long‐PCR) targeting the entire *SERPING1* was also applied^23^. All experiments were performed in the Department of Internal Medicine and Hematology, Research Laboratory.

#### 2.3.2 Genetic methods performed from DNA extracted from dried blood spot

DNA was extracted from dried blood spots on filter cards (CentoCard®) using standard spin column‐based methods. Posteriorly genomic DNA was enzymatically fragmented, and regions of interest were selectively enriched using capture probes targeted against coding regions of a panel of 11 genes (*ADGRE2*, *ANGPT1*, *CPN1*, *F12*, *KNG1*, *NLRP3*, *PLCG2*, *PLG*, *SERPING1*, *SPINK5*, *TNFAIP3*) that may be involved in the pathogenesis of AE episodes. The libraries were generated with Illumina compatible adaptors and sequenced on an Illumina platform to yield an average coverage depth of ∼20X. Raw sequence data analysis, including base calling, demultiplexing, alignment to the hg19 human reference genome (Genome Reference Consortium GRCh37) and variant calling, was performed using a validated in‐house software. In addition, when warranted, analysis of copy number variations (CNVs) was performed using MLPA with the SALSA P243‐B1 probemix provided by MRC‐Holland. Negative cases were further investigated using whole genome sequencing (WGS) leading to the identification of mutations in additional genes related to the phenotype and differential diagnosis for HAE (Supplementary Table S2). All experiments were performed in the Centogene laboratories.

## 3 RESULTS

### 3.1 Demographics

Data from 70 C1‐INH‐HAE (42 female, 28 male, median age: 36.5, (min‐max: 2–75)), 10 C1‐INH‐AAE (5 female, 5 male; median age: 69.5, (min‐max: 45–83)), 6 other AAE (3 female, 3 male; median age: 61, (min‐max: 22–67)) and 3 healthy female (29, 36 and 61 years old) patients were analyzed for the study. Patients were diagnosed at HACRE on the basis of their medical history and the results of complement laboratory tests. In C1‐INH‐HAE type I, diagnosis was established when levels of C4, AgC1‐INH, and fC1‐INH were observed to be low. In the C1‐INH‐HAE type II, C4 levels were low but C1‐INH concentrations were elevated or normal, accompanied by low fC1‐INH, in which case, if the above results were seen, we determined the level of C1q, which is typically normal in the hereditary form and low in the acquired form of C1‐INH deficiency (C1‐INH‐AAE). In the case of C1‐INH‐AAE, the presence of anti‐C1‐INH antibodies also supports the diagnosis (but this is not positive in all patients). If the patient had no family history of AE and complement laboratory tests showed reduced C4, AgC1‐INH, fC1‐IH, C1q was the diagnostic C1‐INH‐AAE. If complement laboratory tests showed no abnormalities and no family history of AE, the diagnosis was other AAE. Comparison of the newly developed DBS‐based LC/MS method to the results of the classical antigenic measurements.

**FIGURE 1:**
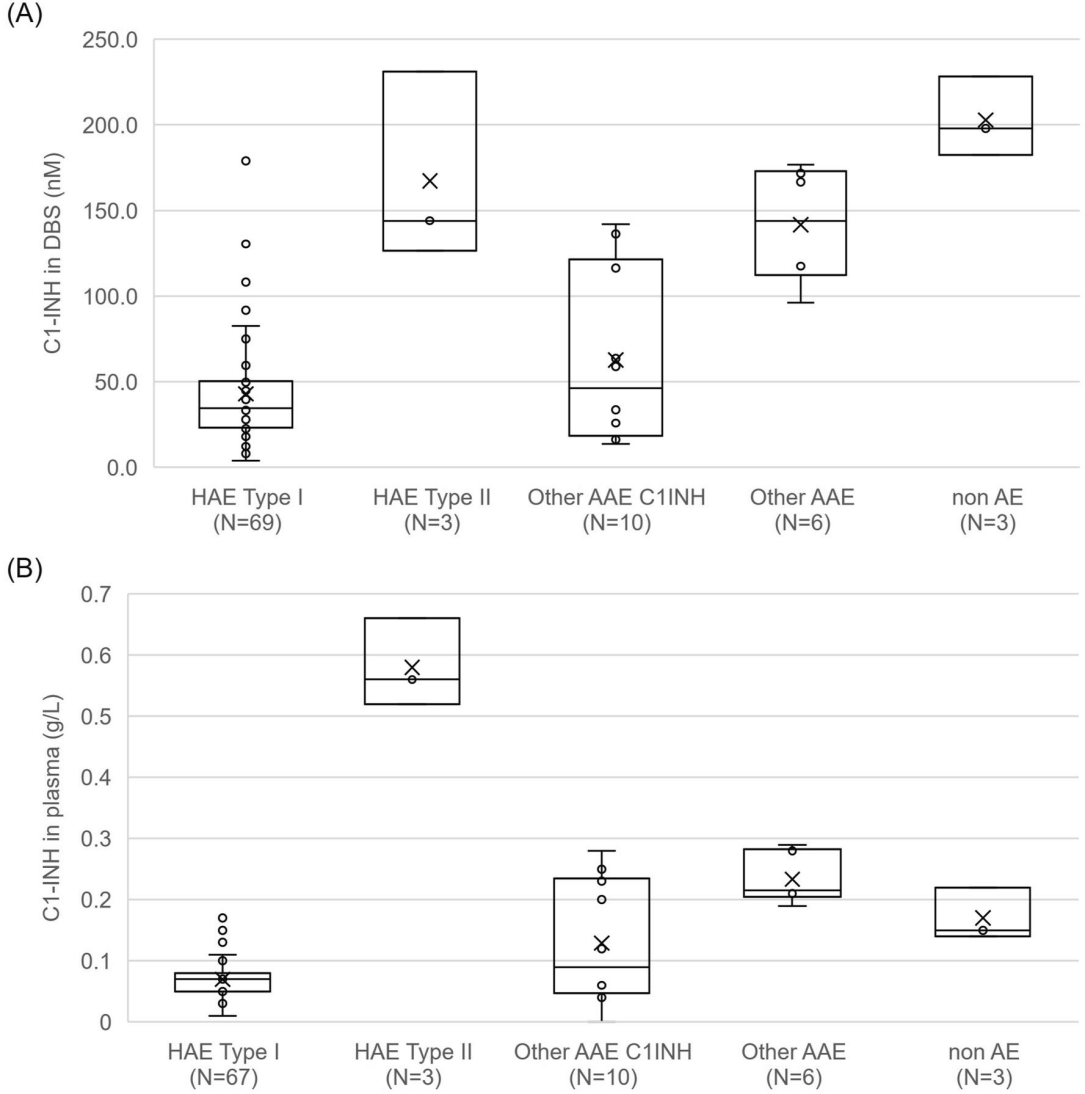
C1‐INH protein levels in dried blood spot (DBS) (A) and plasma (B) in different cohorts included in this study.

**FIGURE 2:**
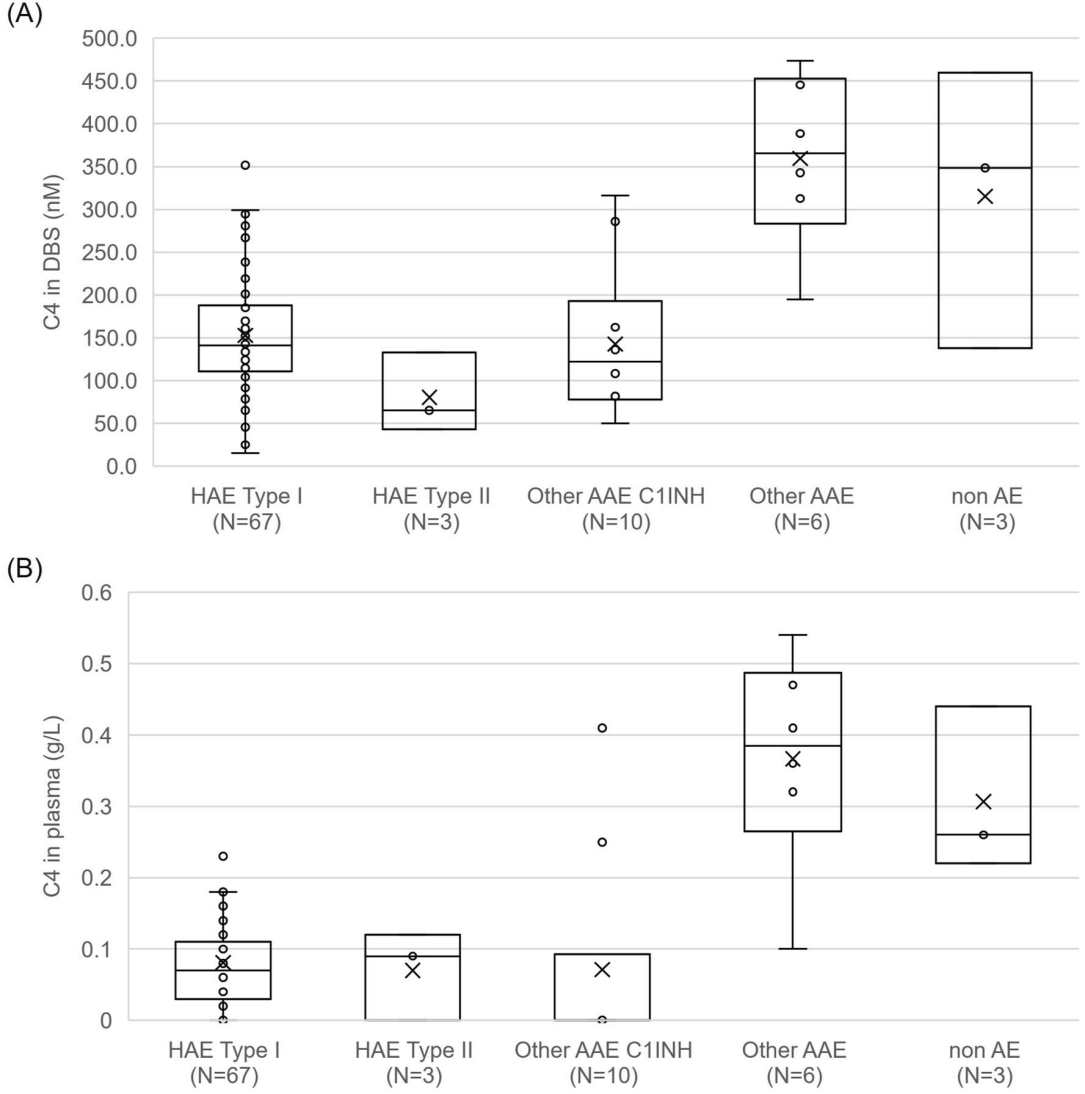
C4 protein levels in dried blood spot (DBS) (A) and plasma (B) in different cohorts included in this study.

The detailed comparison between the classic plasma assays and the new Centogene biomarker analysis from DBS can be viewed in Table 1. Applying the cut‐off values determined previously pathologic C1‐INH were found in 98.5% of the studied HAE I patients (66/67) and 100% of the patients with acquired C1‐inhibitory deficiency (10/10). Complement C4 levels were low in 85% and 80% of the studied C1‐INH‐HAE type I and C1‐INH‐AAE patients, respectively. In the three studied HAE II patients C4 levels were low, while C1‐INH was normal (1/3) or slightly decreased (2/3). Two of the three non‐HAE samples provided normal levels for both C1‐INH and C4, while three of the AE patients (without C1‐INH deficiency) showed normal values in both measurements. A comparison of the distribution of C4 and C1‐INH levels in the studied groups are shown in Figures 1 and 2.

**TABLE 1:** Results of C1‐INH and C4 protein quantification in dried blood spots and plasma.

Patient		Biomarker quantification in DBS					Antibody quantification in plasma				
No.	Cohort	C1‐INH normal values >150 nmol/L		C4 normal values >210 nmol/L		Combined result	C1‐INH normal values >0.15 g/L		C4 normal values >0.15 g/L		Combined result
1	HAE type I	179.0	N	353.6	N	Non HAE	0.08	P	0.23	N	HAE
2	Non AE	197.9	N	459.4	N	Non HAE	0.22	N	0.44	N	Non HAE
3	Non AE	182.5	N	348.4	N	Non HAE	0.15	N	0.26	N	Non HAE
4	HAE type I	94.6	P	351.5	N	HAE	0.05	P	0.23	N	HAE
5	Non AE	228.1	N	138.1	P	HAE	0.14	P	0.22	N	HAE
6	HAE type I	77.9	P	81.0	P	HAE	0.05	P	0.03	P	HAE
7	HAE type I	131.0	P	280.9	N	HAE	0.08	P	0.18	N	HAE
8	HAE type I	19.3	P	99.4	P	HAE	0.05	P	0.04	P	HAE
9	HAE type I	33.3	P	123.4	P	HAE	0.06	P	0.07	P	HAE
10	HAE type I	53.4	P	80.0	P	HAE	0.07	P	0.03	P	HAE
11	HAE type I	36.0	P	71.9	P	HAE	0.03	P	0.02	P	HAE
12	HAE type I	43.4	P	142.7	P	HAE	0.03	P	0.05	P	HAE
13	HAE type I	60.3	P	152.1	P	HAE	0.06	P	0.09	P	HAE
14	HAE type I	36.8	P	133.7	P	HAE	0.03	P	0.09	P	HAE
15	HAE type I	91.7	P	242.5	N	HAE	0.05	P	0.14	P	HAE
16	HAE type I	52.8	P	164.8	P	HAE	0.03	P	0.11	P	HAE
17	HAE type I	40.5	P	141.2	P	HAE	0.07	P	0.1	P	HAE
18	HAE type I	75.0	P	91.5	P	HAE	0.03	P	0.04	P	HAE
19	HAE type I	51.2	P	169.6	P	HAE	0.03	P	0.08	P	HAE
20	HAE type I	49.6	P	104.2	P	HAE	0.06	P	0.03	P	HAE
21	HAE type I	82.5	P	238.5	N	HAE	0.08	P	0.12	P	HAE
22	HAE type I	108.1	P	294.4	N	HAE	0.17	N	0.24	N	Non HAE
23	HAE type I	18.0	P	122.1	P	HAE	0.05	P	0.05	P	HAE
24	HAE type I	22.5	P	78.7	P	HAE	0.1	P	0.1	P	HAE
25	HAE type I	28.0	P	65.3	P	HAE	0.03	P	0.04	P	HAE
26	HAE type I	30.9	P	96.2	P	HAE	0.08	P	0.02	P	HAE
27	HAE type I	30.2	P	45.8	P	HAE	0.07	P	0.01	P	HAE
28	HAE type I	130.4	P	299.4	N	HAE	0.01	P	0.17	N	HAE
29	HAE type I	76.3	P	106.7	P	HAE	0.07	P	0.08	P	HAE
30	HAE type II	231.2	N	132.9	P	HAE	0.66	N	0.12	P	HAE
31	HAE type I	26.7	P	202.3	P	HAE	0.1	P	0.16	N	HAE
32	HAE type I	60.8	P	219.2	N	HAE	0.1	P	0.15	N	HAE
33	HAE type I	29.2	P	71.0	P	HAE	0.07	P	0.06	P	HAE
34	HAE type II	144.0	P	65.4	P	HAE	0.56	N	0.09	P	HAE
35	HAE type I	24.5	P	124.2	P	HAE	0.08	P	0.02	P	HAE
36	HAE type I	3.7	P	25.1	P	HAE	0.11	P	0	P	HAE
37	HAE type I	33.5	P	146.5	P	HAE	0.1	P	0.09	P	HAE
38	HAE type I	47.7	P	123.1	P	HAE	0.13	P	0.06	P	HAE
39	HAE type I	47.4	P	161.5	P	HAE	0.15	N	0.08	P	HAE
40	HAE type I	43.1	P	266.7	N	HAE	0.17	N	0.18	N	Non HAE
41	HAE type I	42.9	P	158.6	P	HAE	0.06	P	0.08	P	HAE
42	HAE type I	27.8	P	114.7	P	HAE	0.06	P	0.03	P	HAE
43	HAE type I	23.2	P	116.3	P	HAE	0.08	P	0.05	P	HAE
44	HAE type I	35.7	P	186.1	P	HAE	0.04	P	0.1	P	HAE
45	HAE type I	37.2	P	175.0	P	HAE	0.06	P	0.07	P	HAE
46	HAE type I	48.1	P	227.5	N	HAE	0.07	P	0.12	P	HAE
47	HAE type I	45.5	P	191.7	P	HAE	0.07	P	0.17	N	HAE
48	HAE type I	11.9	P	15.5	P	HAE	0.06	P	0	P	HAE
49	HAE type I	44.8	P	185.0	P	HAE	0.08	P	0.13	P	HAE
50	HAE type I	63.2	P	188.9	P	HAE	0.07	P	0.09	P	HAE
51	HAE type I	23.2	P	149.6	P	HAE	0.11	P	0.07	P	HAE
52	HAE type I	39.6	P	187.7	P	HAE	0.1	P	0.14	P	HAE
53	HAE type I	23.4	P	137.7	P	HAE	0.08	P	0.02	P	HAE
54	HAE type I	7.9	P	110.7	P	HAE	0.08	P	0.01	P	HAE
55	HAE type I	59.5	P	201.0	P	HAE	0.11	P	0.17	N	HAE
56	HAE type I	18.4	P	155.2	P	HAE	0.07	P	0.06	P	HAE
57	HAE type I	23.5	P	125.6	P	HAE	0.04	P	0.07	P	HAE
58	HAE type I	34.6	P	131.0	P	HAE	0.05	P	0.07	P	HAE
59	HAE type I	12.3	P	147.9	P	HAE	0.04	P	0.03	P	HAE
60	HAE type I	30.6	P	162.3	P	HAE	0.05	P	0.05	P	HAE
61	HAE type II	126.5	P	42.9	P	HAE	0.52	N	0	P	HAE
62	HAE type I	39.6	P	191.5	P	HAE	0.07	P	0.1	P	HAE
63	C1‐INH‐AAE	142.1	P	138.1	P	HAE	0.23	N	0.04	P	HAE
64	HAE type I	22.1	P	124.0	P	HAE	0.04	P	0.03	P	HAE
65	HAE type I	15.2	P	127.5	P	HAE	0.06	P	0.06	P	HAE
66	HAE type I	19.0	P	166.5	P	HAE	0.1	P	0.08	P	HAE
67	HAE type I	19.3	P	124.6	P	HAE	0.06	P	0.03	P	HAE
68	HAE type I	30.0	P	193.3	P	HAE	0.05	P	0.12	P	HAE
69	HAE type I	14.9	P	110.0	P	HAE	0.07	P	0.06	P	HAE
70	HAE type I	20.7	P	117.5	P	HAE	0.06	P	0.04	P	HAE
71	HAE type I	36.0	P	137.3	P	HAE	0.07	P	0.03	P	HAE
72	HAE type I	21.4	P	134.6	P	HAE	0.03	P	0.03	P	HAE
73	HAE type I	24.0	P	160.9	P	HAE	0.05	P	0.07	P	HAE
74	HAE type I	30.3	P	106.2	P	HAE	0.11	P	0.05	P	HAE
75	C1‐INH‐AAE	19.3	P	81.9	P	HAE	0	P	0	P	HAE
76	C1‐INH‐AAE	13.8	P	82.5	P	HAE	0.06	P	0	P	HAE
77	C1‐INH‐AAE	33.5	P	49.8	P	HAE	0.06	P	0	P	HAE
78	Other AAE	121.3	P	445.5	N	HAE	0.19	N	0.47	N	Non HAE
79	Other AAE	176.9	N	473.3	N	Non HAE	0.21	N	0.54	N	Non HAE
80	Other AAE	171.7	N	342.7	N	Non HAE	0.28	N	0.36	N	Non HAE
81	Other AAE	166.7	N	388.6	N	Non HAE	0.29	N	0.41	N	Non HAE
82	C1‐INH‐AAE	25.8	P	65.9	P	HAE	0.04	P	0.01	P	HAE
83	C1‐INH‐AAE	59.0	P	108.1	P	HAE	0.12	P	0	P	HAE
84	C1‐INH‐AAE	16.2	P	136.1	P	HAE	0.05	P	0	P	HAE
85	Other AAE	96.3	P	194.6	P	HAE	0.22	N	0.1	P	HAE
86	C1‐INH‐AAE	63.5	P	285.8	N	HAE	0.20	N	0.25	N	Non HAE
87	C1‐INH‐AAE	136.3	P	162.3	P	HAE	0.28	N	0	P	HAE
88	C1‐INH‐AAE	116.5	P	316.3	N	HAE	0.25	N	0.41	N	Non HAE
89	Other AAE	117.5	P	312.7	N	HAE	0.21	N	0.32	N	Non HAE

Abbreviations: HAE, hereditary angioedema; N, Normal; P, Pathologic.

### 3.2 Comparison of the mutations identified in the two laboratories

In each C1‐INH‐HAE patient, a potentially pathogenic *SERPING1* mutation was identified previously as recently reported.^23^ Genetic analyses from DBS samples were capable of verifying each mutation, both substitutions located in the exonic or intronic regions of *SERPING1* and deletions/duplications ranging from one base up to the whole gene (Table 2, Supplementary Table S2). Additionally, 6 rare mutations have been detected in non HAE patients related to HAE phenotype, *ADGRE2* (c.208 G > A, p.Glu70Lys), *XPNPEP2* (c.1880 C > T, p.Pro627Leu) and *SOX18* (c.919 G > A, p.Glu307Lys), and differential HAE diagnosis, CELSR1 (c.809 A > C, p.His270Pro), VEGFC (c.1062 T > A, p.Asn354Lys) and EPHB4 (c.2711 C > G, p.Pro904Arg) (Supplementary Table S2).

**TABLE 2:** List of SERPING1 (NM_000062.2) variants identified in our study.

DNA change	Protein change	Evidence (PMID)
c.65 C > G	p.Ser22*	15971231^24^
c.94 C > T	p.Gln32*	14635117^25^
c.392_393del	p.Ser131 fs*	14635117^25^
c.435_476del	p.Leu146_Ala159del	14635117^25^
c.550 + 1G > A	p.?	14635117^25^
c.550 + 2dup	p.?	35386643
c.705del	p.Phe236Leufs*2	25258140^26^
c.1029 + 384A > G	p.?	31982983^27^
c.1357_1382dup	p.Ile462Glyfs*123	35386643
c.1396 C > T	p.Arg466Cys	2563376^28^
c.1466del	p.Pro489Leufs*87	23265861^29^
c.1478 G > A	p.Gly493Glu	12402344^30^
c.553 G > C	p.Ala185Pro	25258140^26^
c.596 A > C	p.Tyr199Ser	35386643
c.667 C > T	p.Gln223*	14635117^25^
c.686‐3C > G	p.?	15971231^24^
c.752 T > G	p.Leu251Arg	25258140^26^
c.889 + 1G > A	p.?	35386643
c.988 T > G	p.Tyr330Asp	29753808^31^
c.1480 C > T	p.Arg494*	8755917^32^
c.1493 C > G	p.Pro498Arg	14635117^25^
Deletion exons 1–8 (entire gene)	p.?	11139243^33^; 15971231^24^
Deletion exon 4	p.?	11139243,^33^ among several others
Partial deletion exon 6 (chr11:57,373,584–57,376,415)	p.?	35386643
Deletion of exon 7 and 8	p.?	18758157^34^; 15971231^24^
Duplication of exon 7	p.?	35386643

*Note*: All variants were detected in heterozygous state.

## 4 DISCUSSION

Our results indicate the excellent diagnostic value of a combined biochemical screening and genetic confirmation approach from dried blood samples enabling the local or remote diagnosis of hereditary AE patients.

A biomarker assay was developed for measuring C1‐INH and C4 that can be applied for screening patients with edema symptoms.

In this assay the quantification of antigenic C1‐INH and C4 levels using DBS serves as an initial screening procedure. If any or both markers, C1‐INH or C4, are in the pathological range, the sample is considered potentially pathological and subsequent genetic testing of the sample is recommended. Identifying a (potentially) pathogenic variant in the gene encoding C1‐INH not only confirms the diagnosis of C1‐INH‐HAE but also facilitates the differentiation between type I and type II. Conversely, in cases where both antigenic C1‐INH and C4 levels are normal, the diagnosis of C1‐INH‐HAE can be confidently ruled out, thereby significantly reducing the number of negative cases entering the healthcare system.

While there exists a potential limitation in conducting genetic analyses in all individuals with low C4 levels, this is counteracted by the distinctive edematous phenotype associated with HAE. For instance, ongoing infections or autoimmune diseases such as systemic lupus erythematosus may manifest low C4 levels but without concurrent AE.

In scenarios where the sample is considered as potentially pathological based on DBS measurements but no underlying variant is identified in genetic testing, further investigation (by studying C1‐INH functional activity, C1q level and the presence of anti‐C1‐INH antibodies) at a specialized center is warranted to confirm or exclude the possibility of C1‐INH‐AAE.

In case of the studied samples, the new proteolytic‐based biomarkers showed similar sample distribution as the classical antibody determination for both C1‐INH (Figure 1) and C4 (Figure 2) proteins. The proteolytic biomarker assay in DBS provided only one false negative result in the case of an asymptomatic individual with HAE type I mutation identified through family screening. It should also be noted that samples were obtained from previously diagnosed and treated C1‐INH‐HAE patients, therefore treatment might have influenced C1‐INH level in some cases as reported previously.^35^, ^36^

As expected, from the patients studied by this panel of 11 genes, only the 67 previously diagnosed C1‐INH‐HAE patients carried potentially pathogenic variants of *SERPING1* (Supplementary Table S2). These variations ranged from single nucleotide changes to large deletions/duplications involving even the entire gene. Although single nucleotides or small deletions and insertions changes were the most observed type of variants, a relevant number of patients was also identified with larger CNVs. Of note, 2 patients (father and son) were found to have a partial deletion of exon 6. This CNV was detected due to the next‐generation sequencing (NGS)‐based CNV analysis as MLPA analysis resulted negative in the presence of decreased concentration of C1‐INH. The location of the MLPA probe in the exon 6 was before the start of the deletion, leading to a false‐negative result.^23^ This stresses the importance of the combination of different genetic methods to identify the genetic cause of the disease and provide an accurate diagnosis to the patients.

At last, the use of an extended NGS panel of genes and WGS also allowed us to identify 3 patients with novel rare variants in other genes (*ADGRE2, XPNPEP2* and *SOX18*). These genes are associated with vibratory urticaria, susceptibility to AE induced by angiotensin‐converting enzyme and hypotrichosis‐lymphedema‐telangiectasia syndrome, respectively. However, further studies to evaluate their potential relevance to the patients are necessary.

## 5 CONCLUSIONS

The proposed two‐tier HAE diagnosis, biomarker screening followed by genetic confirmation, based on DBS analysis, proved to work successfully when compared to the classical approach done via immunoassays in liquid plasma. The two laboratories had a 92% agreement on biomarker results and 100% agreement on *SERPING1*mutations. The use of DBS proved to be a versatile and scalable method for testing of genetic diseases like HAE. It enables the measurement and monitoring of patients and their families in remote locations, in a stable matrix, using regular logistics.

## AUTHOR CONTRIBUTIONS

**Zsuzsanna Balla**: Conceptualization (equal); Data curation (equal); Investigation (equal); Writing – original draft (equal); Writing – review & editing (equal). **Marius Ionuţ Iuraşcu**: Conceptualization (equal); Data curation (equal); Methodology (equal); Writing – original draft (equal); Writing – review & editing (equal). **Noemi Andrasi**: Data curation (equal); Writing – review & editing (equal). **Dorottya Csuka**: Formal analysis (equal); Investigation (equal); Methodology (equal); Writing – review & editing (equal). **Lilian Varga**: Project administration (equal); Supervision (equal); Validation (equal); Writing – review & editing (equal). **Agnes Szilagyi**: Formal analysis (equal); Methodology (equal); Validation (equal); Visualization (equal); Writing – review & editing (equal). **Catarina Pereira**: Investigation (equal); Software (equal). **Kornelia Tripolszki**: Formal analysis (equal); Methodology (equal); Software (equal). **Suliman Khan**: Formal analysis (equal); Methodology (equal); Project administration (equal). **Iuliana Susnea**: Formal analysis (equal); Investigation (equal). **Peter Bauer**: Resources (equal); Supervision (equal). **Claudia Cozma**: Resources (equal); Supervision (lead); Writing – review & editing (equal). **Henriette Farkas**: Conceptualization (equal); Resources (equal); Supervision (lead); Writing – review & editing (equal).

## CONFLICT OF INTEREST STATEMENT

Zsuzsanna Balla has participated in clinical trials of CSL Behring, Pharvaris and Takeda. Lilian Varga ‐ has received travel grants from CSL Behring and Shire Human Genetic Therapies Inc. Henriette Farkas has received research grants from CSL Behring, Takeda and Pharming and served as an advisor for these companies and Kalvista and Biocryst, and has participated in clinical trials/registries for BioCryst, CSL Behring, Pharming, Kalvista, Pharvaris and Takeda. The other authors have declared that no conflict of interest exists.

## Supporting information

Supplementary Information S1

## Data Availability

The unpublished data created within this study are available on request to the corresponding author.
