# Supplementary material for: Application of a dried blood spot based proteomic and genetic assay for diagnosing hereditary angioedema
Source: Clin Transl Allergy. 2023 Nov 23;13(11):e12317. doi: 10.1002/clt2.12317 (PMC10668000; doi:10.1002/clt2.12317)
Supplement: Supplementary file 1 — Supplementary Information S1 [file CLT2-13-e12317-s001.docx]

Supplementary Table 1. Clinical details of patients included in the study

| **Patient** | | | | | **Treatment** | **Symptoms** |
| --- | --- | --- | --- | --- | --- | --- |
| **No.** | **Cohort** | **ID** | **Gender** | **Age** |  |  |
| 1 | HAE Type I | HC-001 | M | 44 | Unknown | asymptomatic |
| 2 | non AE | HC-002 | F | 61 | Unknown | asymptomatic |
| 3 | non AE | HC-003 | F | 36 | Unknown | asymptomatic |
| 4 | HAE Type I | HC-004 | F | 74 | Complex scheme | last 5 years: 2 subcutaneous (face) |
| 5 | non AE | HC-005 | F | 29 | Unknown | asymptomatic |
| 6 | HAE Type I | HC-101 | F | 69 | Plasma-derived C1-inhibitor  concentrate | Swelling of the extremities-1991 Swelling of the face-1991 Swelling of the trunk-1991 |
| 7 | HAE Type I | HC-102 | M | 32 | Complex scheme | Swelling of the extremities -1992 Swelling of the trunk-1992 Swelling of the genitals-1992 Abdominal swelling-1992 Abdominal pain-1992 Intestinal edema-1992 |
| 8 | HAE Type I | HC-103 | M | 59 | Complex scheme | Swelling of the extremities -1981 Swelling of the larynx-1981 Swelling of the trunk-1981 Swelling of the genitals-1981 Abdominal swelling-1981 Abdominal pain-1981 Intestinal edema-1981 Respiratory involvement-1981 |
| 9 | HAE Type I | HC-104 | F | 16 | Complex scheme | Swelling of the extremities-2009 Swelling of the face-2009 Swelling of the larynx-2009 Abdominal swelling-2009 Abdominal pain-2009 Intestinal edema-2009 Respiratory involvement-2009 |
| 10 | HAE Type I | HC-105 | F | 44 | Complex scheme | Swelling of the extremities-2009 Swelling of the face-2009 Swelling of the larynx-2009 Abdominal swelling-2009 Abdominal pain-2009 Intestinal edema-2009 Respiratory involvement-2009 |
| 11 | HAE Type I | HC-106 | F | 45 | Complex scheme | Swelling of the extremities-2001 Abdominal swelling-2001 Abdominal pain-2001 Intestinal edema-2001 |
| 12 | HAE Type I | HC-107 | F | 20 | Complex scheme | Swelling of the extremities - 2004 Swelling of the larynx - 2004 Respiratory involvement - 2004 |
| 13 | HAE Type I | HC-108 | M | 29 | Complex scheme | Swelling of the extremities - 1993 Swelling of the face - 1993 Abdominal swelling - 1993 Abdominal pain - 1993 Intestinal edema - 1993 Respiratory involvement - 1993 |
| 14 | HAE Type I | HC-109 | F | 7 | Complex scheme | Swelling of the extremities-2014 Swelling of the trunk-2014 Repetitive nausea-2014 |
| 15 | HAE Type I | HC-110 | M | 9 | Plasma-derived C1-inhibitor  concentrate | asymptomatic |
| 16 | HAE Type I | HC-111 | F | 41 | Plasma-derived C1-inhibitor  concentrate | asymptomatic |
| 17 | HAE Type I | HC-112 | F | 40 | Complex scheme | Swelling of the extremities - 1995 Swelling of the face - 1995 Swelling of the larynx - 1995 Swelling of the trunk - 1995 Swelling of the genitals - 1995 Abdominal swelling - 1995 Abdominal pain - 1995 Intestinal edema - 1995 Respiratory involvement - 1995 |
| 18 | HAE Type I | HC-113 | F | 25 | Complex scheme | Swelling of the extremities - 1999 Swelling of the larynx - 1999 Swelling of the trunk - 1999 Swelling of the genitals - 1999 Abdominal swelling - 1999 Intestinal edema - 1999 |
| 19 | HAE Type I | HC-114 | M | 31 | Complex scheme | Swelling of the extremities - 1999 Swelling of the face - 1999 Swelling of the trunk - 1999 Abdominal swelling - 1999 Abdominal pain - 1999 Repetitive diarrhea - 1999 |
| 20 | HAE Type I | HC-115 | F | 52 | Complex scheme | Swelling of the extremities - 1989 Swelling of the face - 1989 Swelling of the larynx - 1989 Swelling of the trunk - 1989 Abdominal swelling - 1989 Abdominal pain - 1989 Intestinal edema - 1989 Respiratory involvement - 1989 |
| 21 | HAE Type I | HC-116 | M | 50 | Complex scheme | in the last 5 years asymptomatic |
| 22 | HAE Type I | HC-117 | M | 21 | Complex scheme | Swelling of the extremities - 2001 Swelling of the face - 2001 Swelling of the trunk - 2001 |
| 23 | HAE Type I | HC-118 | F | 64 | Complex scheme | 25 HAE in the last five years: subcutaneous, larynx, abdominal, face |
| 24 | HAE Type I | HC-119 | F | 2 | Plasma-derived C1-inhibitor  concentrate | asymptomatic |
| 25 | HAE Type I | HC-120 | F | 5 | Plasma-derived C1-inhibitor  concentrate | Swelling of the face - 2015 |
| 26 | HAE Type I | HC-121 | F | 37 | Complex scheme | Swelling of the extremities - 1999 Swelling of the tonge - 1999 Swelling of the trunk - 1999 Abdominal swelling - 1999 Abdominal pain - 1999 Repetitive diarrhea - 1999 Intestinal Edema - 1999 |
| 27 | HAE Type I | HC-122 | F | 25 | Complex scheme | 26 HAE attack in the last five years: abdominal |
| 28 | HAE Type I | HC-123 | F | 48 | Complex scheme | 1 HAE attack in the last five years: lip |
| 29 | HAE Type I | HC-124 | F | 24 | Complex scheme | Swelling of the extremities - 1998 Swelling of the face - 1998 Swelling of the trunk - 1998 Abdominal swelling - 1998 Abdominal pain - 1998 Intestinal Edema - 1998 |
| 30 | HAE Type II | HC-125 | M | 7 | Plasma-derived C1-inhibitor  concentrate | Swelling of the extremities - 2013 Swelling of the trunk - 2013 |
| 31 | HAE Type I | HC-126 | M | 24 | Complex scheme | Swelling of the extremities - 1998 Swelling of the face - 1998 Swelling of the tongue - 1998 Swelling of the larynx - 1998 Swelling of the trunk - 1998 Swelling of the genitals - 1998 Abdominal swelling - 1998 Abdominal pain - 1998 Intestinal edema - 1998 Respiratory involvement - 1998 |
| 32 | HAE Type I | HC-127 | F | 10 | Plasma-derived C1-inhibitor  concentrate | asymptomatic |
| 33 | HAE Type I | HC-128 | F | 48 | Complex scheme | Swelling of the extremities - 1998 Swelling of the face - 1998 Swelling of the larynx - 1998 Swelling of the trunk - 1998 Swelling of the genitals - 1998 Abdominal swelling - 1998 Abdominal pain - 1998 Intestinal edema - 1998 Respiratory involvement - 1998 |
| 34 | HAE Type II | HC-129 | F | 36 | Plasma-derived C1-inhibitor  concentrate | Swelling of the extremities - 1996 Swelling of the larynx - 1996 Swelling of the trunk - 1996 Abdominal swelling - 1996 Abdominal pain - 1996 Intestinal edema - 1996 |
| 35 | HAE Type I | HC-130 | F | 32 | Complex scheme | Swelling of the extremities - 1997 Swelling of the trunk - 1997 Abdominal swelling - 1997 Abdominal pain - 1997 Intestinal edema - 1997 |
| 36 | HAE Type I | HC-132 | F | 75 | Complex scheme | Swelling of the extremities - 1991 Swelling of the face - 1991 Swelling of the tongue - 1991 Swelling of the larynx - 1991 Swelling of the trunk - 1991 Respiratory involvement - 1991 |
| 37 | HAE Type I | HC-133 | F | 53 | Complex scheme | Swelling of the extremities - 2002 Swelling of the trunk - 2002 Abdominal swelling - 2002 Abdominal pain - 2002 Intestinal edema - 2002 |
| 38 | HAE Type I | HC-135 | F | 16 | Plasma-derived C1-inhibitor  concentrate | asymptomatic |
| 39 | HAE Type I | HC-136 | F | 9 | Plasma-derived C1-inhibitor  concentrate | asymptomatic |
| 40 | HAE Type I | HC-137 | M | 57 | Complex scheme | Swelling of the extremities - 2002 Swelling of the face - 2002 Swelling of the trunk - 2002 |
| 41 | HAE Type I | HC-138 | M | 19 | Complex scheme | Swelling of the extremities - 2002 Swelling of the face - 2002 Swelling of the trunk - 2002 Abdominal swelling - 2002 Abdominal pain - 2002 Intestinal edema - 2002 |
| 42 | HAE Type I | HC-139 | M | 16 | Plasma-derived C1-inhibitor  concentrate | Swelling of the extremities - 2006 |
| 43 | HAE Type I | HC-140 | F | 21 | Complex scheme | Swelling of the extremities Swelling of the face -2005 Swelling of the larynx -2005 Swelling of the trunk -2005 Abdominal swelling -2005 Abdominal pain -2005 Intestinal edema -2005 Respiratory involvement -2005 |
| 44 | HAE Type I | HC-141 | M | 65 | Complex scheme | Swelling of the extremities - 2005 Swelling of the larynx - 2005 Swelling of the trunk - 2005 Swelling of the genitals - 2005 Abdominal swelling - 2005 Abdominal pain - 2005 Intestinal edema - 2005 Respiratory involvement - 2005 |
| 45 | HAE Type I | HC-142 | F | 49 | Complex scheme | Swelling of the extremities - 2001 Swelling of the larynx - 2001 Swelling of the trunk - 2001 Swelling of the genitals - 2001 Abdominal swelling - 2001 Abdominal pain - 2001 Intestinal edema - 2001 Respiratory involvement - 2001 |
| 46 | HAE Type I | HC-143 | M | 33 | Complex scheme | Swelling of the extremities - 1997 Swelling of the trunk - 1997 Abdominal swelling - 1997 Abdominal pain - 1997 Intestinal edema - 1997 |
| 47 | HAE Type I | HC-144 | M | 52 | Complex scheme | Swelling of the extremities - 1999 Swelling of the trunk - 1999 |
| 48 | HAE Type I | HC-145 | F | 22 | Complex scheme | Swelling of the extremities - 2006 Swelling of the face - 2006 Swelling of the tongue - 2006 Swelling of the larynx - 2006 Swelling of the trunk - 2006 Swelling of the genitals - 2006 Abdominal swelling - 2006 Abdominal pain - 2006 Intestinal edema - 2006 Respiratory involvement - 2006 |
| 49 | HAE Type I | HC-146 | F | 41 | Complex scheme | Swelling of the extremities - 2018 Swelling of the face - 2018 Swelling of the trunk - 2018 Abdominal swelling - 2018 Abdominal pain - 2018 Intestinal edema - 2018 |
| 50 | HAE Type I | HC-147 | M | 55 | Complex scheme | Swelling of the extremities - 2007 |
| 51 | HAE Type I | HC-148 | F | 33 | Complex scheme | Swelling of the extremities - 2005 Abdominal swelling - 2005 Abdominal pain - 2005 Intestinal edema - 2005 |
| 52 | HAE Type I | HC-149 | M | 56 | Complex scheme | Swelling of the extremities -2005 |
| 53 | HAE Type I | HC-150 | M | 35 | Complex scheme | Swelling of the extremities Swelling of the larynx Abdominal swelling  Abdominal pain Intestinal edema |
| 54 | HAE Type I | HC-151 | M | 63 | Complex scheme | Swelling of the extremities - 1995 Swelling of the face - 1995 Swelling of the larynx - 1995 Abdominal swelling - 1995 Intestinal edema - 1995 Respiratory involvement - 1995 |
| 55 | HAE Type I | HC-152 | F | 50 | Complex scheme | asymptomatic |
| 56 | HAE Type I | HC-153 | M | 27 | Complex scheme | Swelling of the extremities - 2010 Swelling of the face - 2010 Swelling of the larynx - 2010 Swelling of the genitals - 2010 Abdominal swelling - 2010 Abdominal pain - 2010 Intestinal edema - 2010 |
| 57 | HAE Type I | HC-154 | F | 31 | Complex scheme | Swelling of the extremities - 2010 Abdominal swelling - 2010 Abdominal pain - 2010 Intestinal edema - 2010 |
| 58 | HAE Type I | HC-155 | M | 23 | Complex scheme | 43 HAE attack in the last five years: subcutaneous, larynx, abdominal, face, genital |
| 59 | HAE Type I | HC-156 | M | 52 | Complex scheme | in every 2-3 month HAE attack: subcutaneous, abdominal, genital |
| 60 | HAE Type I | HC-157 | M | 21 | Complex scheme | yearly 3-4 times an HAE attack: genital, abdominal, subcutaneous, larynx |
| 61 | HAE Type II | HC-158 | F | 46 | Complex scheme | 17 HAE attack in the last five years: subcutaneous, abdominal, face |
| 62 | HAE Type I | HC-159 | F | 58 | Complex scheme | 11 HAE attack in the last five years: subcutaneous, abdominal |
| 63 | C1-INH-AAE | HC-160 | M | 77 | Complex scheme | 3 AE attacks in the last five years: face, larynx |
| 64 | HAE Type I | HC-161 | M | 14 | Complex scheme | 9 HAE attack in the last five years: subcutaneous, face, abdominal |
| 65 | HAE Type I | HC-162 | M | 46 | Complex scheme | 41 HAE attack in the last five years: subcutaneous, face, abdominal, genitals |
| 66 | HAE Type I | HC-163 | F | 64 | Complex scheme | 14 HAE attack in the last five years: subcutenous |
| 67 | HAE Type I | HC-164 | F | 39 | Complex scheme | 31 HAE attack in the last five years: subcutaneous, larynx, abdominal, face |
| 68 | HAE Type I | HC-165 | M | 60 | Complex scheme | 20 HAE attack in the last five years: subcutaneous, abdominal |
| 69 | HAE Type I | HC-166 | F | 46 | Complex scheme | 12 HAE attack in the last five year: larynx, abdominal |
| 70 | HAE Type I | HC-167 | F | 68 | Complex scheme | 1 HAE attack in the last five years: subcutaneous |
| 71 | HAE Type I | HC-168 | F | 16 | Complex scheme | 6 HAE attack in the last five years: subcutaneous |
| 72 | HAE Type I | HC-169 | F | 49 | Complex scheme | 51 HAE attacks in the last five years: subcutaneous, larynx, abdominal |
| 73 | HAE Type I | HC-170 | M | 31 | Complex scheme | 30 HAE attacks in the last five years: subcutaneous, larynx, abdominal |
| 74 | HAE Type I | HC-171 | F | 24 | Complex scheme | 41 HAE attack in the last five years: subcutaneous, abdominal |
| 75 | C1-INH-AAE | HC-172 | F | 68 | Complex scheme | 31 AE attacks in the last five years: subcutaneous, abdominal, genitals |
| 76 | C1-INH-AAE | HC-601 | M | 58 | Complex scheme | 19 AE attacks in the last five years: subcutaneous, genitals, abdominals, tongue |
| 77 | C1-INH-AAE | HC-602 | F | 71 | Complex scheme | 9 AE attacks in the last five years: subcutaneous, larynx, abdominal |
| 78 | Other AAE | HC-603 | M | 61 | Unknown | permanent fluctual laryngeal edema |
| 79 | Other AAE | HC-604 | M | 61 | Unknown | permanent fluctual laryngeal edema |
| 80 | Other AAE | HC-605 | F | 22 | Unknown | permanent fluctual laryngeal edema |
| 81 | Other AAE | HC-606 | M | 61 | Unknown | permanent fluctual laryngeal edema |
| 82 | C1-INH-AAE | HC-607 | F | 71 | Complex scheme | 41 HAE attack in the last five years: subcutaneous, face, abdominal, genitals |
| 83 | C1-INH-AAE | HC-608 | F | 83 | Complex scheme | 10 AE attack in the last five years: subcutaneous, face, larynx |
| 84 | C1-INH-AAE | HC-609 | M | 45 | Complex scheme | 97 AE attacks in the last five years: subcutaneous, abdominal, larynx, face |
| 85 | Other AAE | HC-610 | F | 67 | Complex scheme | Swelling of the extremities - 1985 |
| 86 | C1-INH-AAE | HC-611 | M | 61 | Complex scheme | Swelling of the extremities – 2007, 2017 Swelling of the upper airway and face – 2017 Swelling of the eyelid - 2017 |
| 87 | C1-INH-AAE | HC-612 | F | 57 | Complex scheme | 42 AAE attack in the last five years: subcutaneous, face, abdominal, genital, upper airways |
| 88 | C1-INH-AAE | HC-613 | M | 73 | Complex scheme | 10 AAE attack in the last five years: subcutaneous, face, lips, tongue |
| 89 | Other AAE | HC-614 | F | 64 | Complex scheme | 70 AAE attack in the last five years: subcutaneous, face, abdominal, tongue, upper airways |

Supplementary Table 2. Genetic workup of the patients included in the study

| **No.** | **Cohort** | **Patient** | **Gene** | **SNV/CNV Position** | **Protein change** | **SNV/CNV classification** | **Zygosity** | **Evidence (PMID)** |
| --- | --- | --- | --- | --- | --- | --- | --- | --- |
| 1 | HAE Type I | HC-001 | SERPING 1 | c.686-3C>G | p.? | Class 1 - Pathogenic | heterozygous | 15971231 |
| 2 | non AE | HC-002 | CELSR1 | c.809A>C | p.His270Pro | Class 3 - VUS | heterozygous |  |
| 3 | non AE | HC-003 | CELSR1 | c.809A>C, c.9036-187G>A | p.His270Pro | Class 3 - VUS | heterozygous |  |
| 4 | HAE Type I | HC-004 | SERPING 1 | c.686-3C>G | p.? | Class 1 - Pathogenic | heterozygous | 15971231 |
| 5 | non AE | HC-005 | VEGFC | c.1062T>A | p.Asn354Lys | Class 3 - VUS | heterozygous |  |
| 6 | HAE Type I | HC-101 | SERPING 1 | c.553G>C | p.Ala185Pro | Class 1 - Pathogenic | heterozygous | 25258140 |
| 7 | HAE Type I | HC-102 | SERPING 1 | del whole gene (exon 1 to 8) | p.? | Class 1 - Pathogenic | heterozygous | 11139243; 15971231 |
| 8 | HAE Type I | HC-103 | SERPING 1 | del whole gene (exon 1 to 8) | p.? | Class 1 - Pathogenic | heterozygous | 11139243; 15971231 |
| 9 | HAE Type I | HC-104 | SERPING 1 | c.752T>G | p.Leu251Arg | Class 1 - Pathogenic | heterozygous | 25258140 |
| 10 | HAE Type I | HC-105 | SERPING 1 | c.752T>G | p.Leu251Arg | Class 1 - Pathogenic | heterozygous | 25258140 |
| 11 | HAE Type I | HC-106 | SERPING 1 | c.553G>C | p.Ala185Pro | Class 1 - Pathogenic | heterozygous | 25258140 |
| 12 | HAE Type I | HC-107 | SERPING 1 | c.553G>C | p.Ala185Pro | Class 1 - Pathogenic | heterozygous | 25258140 |
| 13 | HAE Type I | HC-108 | SERPING 1 | c.435_476del | p.Leu146_Ala159del | Class 1 - Pathogenic | heterozygous | 14635117 |
| 14 | HAE Type I | HC-109 | SERPING 1 | c.889+1G>A | p.? | Class 1 - Pathogenic | heterozygous | 35386643 |
| 15 | HAE Type I | HC-110 | SERPING 1 | c.889+1G>A | p.? | Class 1 - Pathogenic | heterozygous | 35386643 |
| 16 | HAE Type I | HC-111 | SERPING 1 | c.889+1G>A | p.? | Class 1 - Pathogenic | heterozygous | 35386643 |
| 17 | HAE Type I | HC-112 | SERPING 1 | del exon 7 to 8 | p.? | Class 1 - Pathogenic | heterozygous | 18758157; 15971231 |
| 18 | HAE Type I | HC-113 | SERPING 1 | c.65C>G | p.Ser22* | Class 1 - Pathogenic | heterozygous | 15971231 |
| 19 | HAE Type I | HC-114 | SERPING 1 | del whole gene (exon 4) | p.? | Class 1 - Pathogenic | heterozygous | 11139243, among several others |
| 20 | HAE Type I | HC-115 | SERPING 1 | del whole gene (exon 4) | p.? | Class 1 - Pathogenic | heterozygous | 11139243, among several others |
| 21 | HAE Type I | HC-116 | SERPING 1 | c.389G>A | p.Cys130Tyr | Class 3 - VUS | heterozygous |  |
| 22 | HAE Type I | HC-117 | SERPING 1 | c.389G>A | p.Cys130Tyr | Class 3 - VUS | heterozygous |  |
| 23 | HAE Type I | HC-118 | SERPING 1 | c.667C>T | p.Gln223* | Class 1 - Pathogenic | heterozygous | 14635117 |
| 24 | HAE Type I | HC-119 | SERPING 1 | c.(550+2dup) | p.? | Class 1 - Pathogenic | heterozygous | 35386643 |
| 25 | HAE Type I | HC-120 | SERPING 1 | c.435_476del | p.Leu146_Ala159del | Class 1 - Pathogenic | heterozygous | 14635117 |
| 26 | HAE Type I | HC-121 | SERPING 1 | del whole gene (exon 1 to 8) | p.? | Class 1 - Pathogenic | heterozygous | 11139243; 15971231 |
| 27 | HAE Type I | HC-122 | SERPING 1 | c.435_476del | p.Leu146_Ala159del | Class 1 - Pathogenic | heterozygous | 14635117 |
| 28 | HAE Type I | HC-123 | SERPING 1 | c.686-3C>G | p.? | Class 1 - Pathogenic | heterozygous | 15971231 |
| 29 | HAE Type I | HC-124 | SERPING 1 | deep intronic variant c.1029+384A>G | p.? | Class 1 - Pathogenic | heterozygous | 31982983 |
| 30 | HAE Type II | HC-125 | SERPING 1 | c.1396C>T | p.Arg466Cys | Class 1 - Pathogenic | heterozygous | 2563376 |
| 31 | HAE Type I | HC-126 | SERPING 1 | c.550+1G>A | p.? | Class 1 - Pathogenic | heterozygous | 14635117 |
| 32 | HAE Type I | HC-127 | SERPING 1 | c.550+1G>A | p.? | Class 1 - Pathogenic | heterozygous | 14635117 |
| 33 | HAE Type I | HC-128 | SERPING 1 | c.550+1G>A | p.? | Class 1 - Pathogenic | heterozygous | 14635117 |
| 34 | HAE Type II | HC-129 | SERPING 1 | c.1396C>T | p.Arg466Cys | Class 1 - Pathogenic | heterozygous | 2563376 |
| 35 | HAE Type I | HC-130 | SERPING 1 | het deletion exon 4 | p.? | Class 1 - Pathogenic | heterozygous | 11139243, among several others |
| 36 | HAE Type I | HC-132 | SERPING 1 | het deletion exon 4 | p.? | Class 1 - Pathogenic | heterozygous | 11139243, among several others |
| 37 | HAE Type I | HC-133 | SERPING 1 | c.94C>T | p.Gln32* | Class 1 - Pathogenic | heterozygous | 14635117 |
| 38 | HAE Type I | HC-135 | SERPING 1 | c.435_476del | p.Leu146_Ala159del | Class 1 - Pathogenic | heterozygous | 14635117 |
| 39 | HAE Type I | HC-136 | SERPING 1 | c.435_476del | p.Leu146_Ala159del | Class 1 - Pathogenic | heterozygous | 14635117 |
| 40 | HAE Type I | HC-137 | SERPING 1 | c.94C>T | p.Gln32* | Class 1 - Pathogenic | heterozygous | 14635117 |
| 41 | HAE Type I | HC-138 | SERPING 1 | c.435_476del | p.Leu146_Ala159del | Class 1 - Pathogenic | heterozygous | 14635117 |
| 42 | HAE Type I | HC-139 | SERPING 1 | het dup. exon 7 | p.? | Class 1 - Pathogenic | heterozygous | 35386643 |
| 43 | HAE Type I | HC-140 | SERPING 1 | het dup. exon 7 | p.? | Class 1 - Pathogenic | heterozygous | 35386643 |
|  |  |  | EPHB4 | c.2711C>G | p.Pro904Arg | Class 3 - VUS | heterozygous |  |
| 44 | HAE Type I | HC-141 | SERPING 1 | het dup. exon 7 | p.? | Class 1 - Pathogenic | heterozygous | 35386643 |
| 45 | HAE Type I | HC-142 | SERPING 1 | c.988T>G | p.Tyr330Asp | Class 1 - Pathogenic | heterozygous | 29753808 |
| 46 | HAE Type I | HC-143 | SERPING 1 | c. 1357_1382dup | p.Ile462Glyfs*123 | Class 2 - Likely pathogenic | heterozygous | 35386643 |
| 47 | HAE Type I | HC-144 | SERPING 1 | c.1493C>G | p.Pro498Arg | Class 2 - Likely pathogenic | heterozygous | 14635117 |
| 48 | HAE Type I | HC-145 | SERPING 1 | c.435_476del | p.Leu146_Ala159del | Class 1 - Pathogenic | heterozygous | 14635117 |
| 49 | HAE Type I | HC-146 | SERPING 1 | c.686-3C>G | p.? | Class 1 - Pathogenic | heterozygous | 15971231 |
| 50 | HAE Type I | HC-147 | SERPING 1 | c.435_476del | p.Leu146_Ala159del | Class 1 - Pathogenic | heterozygous | 14635117 |
| 51 | HAE Type I | HC-148 | SERPING 1 | c.1466del | p.Pro489Leufs*87 | Class 1 - Pathogenic | heterozygous | 23265861 |
| 52 | HAE Type I | HC-149 | SERPING 1 | c.1466del | p.Pro489Leufs*87 | Class 1 - Pathogenic | heterozygous | 23265861 |
| 53 | HAE Type I | HC-150 | SERPING 1 | c.1478G>A | p.Gly493Glu | Class 1 - Pathogenic | heterozygous | 12402344 |
| 54 | HAE Type I | HC-151 | SERPING 1 | del whole gene (exon 1 to 8) | p.? | Class 1 - Pathogenic | heterozygous | 11139243; 15971231 |
| 55 | HAE Type I | HC-152 | SERPING 1 | c.686-3C>G | p.? | Class 1 - Pathogenic | heterozygous | 15971231 |
| 56 | HAE Type I | HC-153 | SERPING 1 | c.705del | p.Phe236Leufs*2 | Class 1 - Pathogenic | heterozygous | 25258140 |
| 57 | HAE Type I | HC-154 | SERPING 1 | c.705del | p.Phe236Leufs*2 | Class 1 - Pathogenic | heterozygous | 25258140 |
| 58 | HAE Type I | HC-155 | SERPING 1 | c.392_393del | p.Ser131fs* | Class 1 - Pathogenic | heterozygous | 14635117 |
| 59 | HAE Type I | HC-156 | SERPING 1 | partial del. exon 6 | p.? | Class 1 - Pathogenic | heterozygous | 35386643 |
| 60 | HAE Type I | HC-157 | SERPING 1 | partial del. exon 6 | p.? | Class 1 - Pathogenic | heterozygous | 35386643 |
| 61 | HAE Type II | HC-158 | SERPING 1 | c.1396C>T | p.Arg466Cys | Class 1 - Pathogenic | heterozygous | 2563376 |
| 62 | HAE Type I | HC-159 | SERPING 1 | c.435_476del | p.Leu146_Ala159del | Class 1 - Pathogenic | heterozygous | 14635117 |
| 63 | C1-INH-AAE | HC-160 | XPNPEP2 | c.1880C>T | p.Pro627Leu | Class 3 - VUS | Hemizygous |  |
| 64 | HAE Type I | HC-161 | SERPING 1 | c.1480C>T | p.Arg494* | Class 1 - Pathogenic | heterozygous | 8755917 |
| 65 | HAE Type I | HC-162 | SERPING 1 | c.1480C>T | p.Arg494* | Class 1 - Pathogenic | heterozygous | 8755917 |
| 66 | HAE Type I | HC-163 | SERPING 1 | c.435_476del | p.Leu146_Ala159del | Class 1 - Pathogenic | heterozygous | 14635117 |
| 67 | HAE Type I | HC-164 | SERPING 1 | c.435_476del | p.Leu146_Ala159del | Class 1 - Pathogenic | heterozygous | 14635117 |
| 68 | HAE Type I | HC-165 | SERPING 1 | c.435_476del | p.Leu146_Ala159del | Class 1 - Pathogenic | heterozygous | 14635117 |
| 69 | HAE Type I | HC-166 | SERPING 1 | c.94C>T | p.Gln32* | Class 1 - Pathogenic | heterozygous | 14635117 |
| 70 | HAE Type I | HC-167 | SERPING 1 | c.94C>T | p.Gln32* | Class 1 - Pathogenic | heterozygous | 14635117 |
| 71 | HAE Type I | HC-168 | SERPING 1 | c.94C>T | p.Gln32* | Class 1 - Pathogenic | heterozygous | 14635117 |
| 72 | HAE Type I | HC-169 | SERPING 1 | c.65C>G | p.Ser22* | Class 1 - Pathogenic | heterozygous | 15971231 |
| 73 | HAE Type I | HC-170 | SERPING 1 | c.94C>T | p.Gln32* | Class 1 - Pathogenic | heterozygous | 14635117 |
| 74 | HAE Type I | HC-171 | SERPING 1 | c.596A>C | p.Tyr199Ser | Class 1 - Pathogenic | heterozygous | 35386643 |
| 75 | C1-INH-AAE | HC-172 | negative |  |  |  |  |  |
| 76 | C1-INH-AAE | HC-601 | negative |  |  |  |  |  |
| 77 | C1-INH-AAE | HC-602 | negative |  |  |  |  |  |
| 78 | Other AAE | HC-603 | negative |  |  |  |  |  |
| 79 | Other AAE | HC-604 | negative |  |  |  |  |  |
| 80 | Other AAE | HC-605 | negative |  |  |  |  |  |
| 81 | Other AAE | HC-606 | negative |  |  |  |  |  |
| 82 | C1-INH-AAE | HC-607 | negative |  |  |  |  |  |
| 83 | C1-INH-AAE | HC-608 | negative |  |  |  |  |  |
| 84 | C1-INH-AAE | HC-609 | negative |  |  |  |  |  |
| 85 | Other AAE | HC-610 | SOX18 | c.919G>A | p.Glu307Lys | unclear | heterozygous |  |
| 86 | C1-INH-AAE | HC-611 | negative |  |  |  |  |  |
| 87 | C1-INH-AAE | HC-612 | negative |  |  |  |  |  |
| 88 | C1-INH-AAE | HC-613 | ADGRE2 | c.208G>A | p.Glu70Lys | Class 3 - VUS | heterozygous |  |
| 89 | Other AAE | HC-614 | negative |  |  |  |  |  |
